# Pediatric Surgeon Perceptions of Participation in External Patient Safety Programs: impact on Patient Safety

**DOI:** 10.1097/pq9.0000000000000124

**Published:** 2018-12-06

**Authors:** Loren Berman, Shawn Rangel, KuoJen Tsao

**Affiliations:** From the * Department of Surgery, Nemours/Alfred I. duPont Hospital for Children, Wilmington, Del.; † Department of Surgery, Sidney Kimmel College at Thomas Jefferson University, Philadelphia, Pa.; ‡ Boston Children’s Hospital, Harvard Medical School, Boston, Mass.; § Department of Surgery, University of Texas Houston-Memorial Hermann Center for Healthcare Quality and Safety, Houston, Tex.

## Abstract

**Introduction:**

Surgeons play a crucial role in preventing harm and contributing to the safety culture of their institutions. External safety data programs are designed to review adverse events and provide performance benchmarks to ameliorate future adverse events. The extent to which pediatric surgeons are aware of these programs, utilize data from these programs, and believe that they improve patient safety, is unknown.

**Methods:**

A cross-sectional survey of the American Pediatric Surgical Association membership was conducted to evaluate participation in and attitudes toward national safety benchmark programs (eg, National Surgical Quality Improvement Program). Surgeons’ perceptions of these activities, including barriers to participation and utilization of safety data, were measured. We performed standard frequency analyses and tests of associations between surgeon characteristics and the likelihood of utilizing safety data.

**Results:**

The response rate was 38% (353/928). Seventy-two percentage of respondents reported institutional participation in external safety benchmark programs. Of those, only 68% utilized data to improve or monitor patient safety. Surgeon-reported barriers to this process included lack of knowledge, time, and institutional resources.

**Conclusions:**

Many pediatric surgeons are aware of institutional participation in external safety data programs, but only a portion are involved in the utilization of these data. We have identified several barriers to participation and data utilization. These findings can help direct educational efforts to optimize our ability to learn from adverse event benchmarking and improve pediatric surgical care.

## INTRODUCTION

In recent years, external safety benchmark data have been used to assess performance improvement in surgery. Surgical patient data collection registries such as the American College of Surgeons–National Surgical Quality Improvement Program (NSQIP) can facilitate a more broad-scale, scientific approach to adverse event review with external benchmark comparison. By utilizing trained surgical clinical reviewers to extract data from patient charts and standardizing definitions of complications and patient comorbidities, American College of Surgeons–NSQIP assists participating institutions in tracking the incidence of adverse events and monitoring the impact of quality improvement projects.^1^

Some reports suggest that participation in external patient safety programs results in improved outcomes,^2,3^ while others do not support this finding.^4^ Overall, the effectiveness of these endeavors remains unproven, leading to skepticism in their utility. Moreover, many surgeons may work at institutions that do not participate in external patient safety programs, and providers at participating institutions often do not have access to compiled data or may not understand how to use it effectively.

Although participation in external patient safety programs can help to improve patient safety, it is unclear to what extent pediatric surgeons participate in these activities, what barriers compromise participation, and to what degree pediatric surgeons believe that they prevent harm. We describe the results of a survey of pediatric surgeons assessing their participation in and attitudes toward external patient safety programs.

## METHODS

### Study Design

Active members of the American Pediatric Surgery Association (APSA) were invited to participate in an online cross-sectional survey about their perceptions and practices in patient safety. We collected demographic information and practice setting, including whether respondents held leadership, education, or safety roles within their institutions. The survey included descriptive questions about whether their affiliated institutions used or reported data to external safety programs. An iterative process based on thematic audit and feedback developed the survey questions by the APSA Quality and Safety Committee. The survey included Likert-scale, multiple choice, and dichotomous answer choices, in addition to open-ended questions.

### Statistical Analysis

Standard frequency analyses and chi-square/Fisher’s exact test to evaluate associations between respondent characteristics and survey responses were performed. Likert scale responses were dichotomized to agree/strongly agree versus neutral/disagree/strongly disagree. Content analysis was used to summarize open-ended response questions.^5^ All research procedures were approved through exemption status by The University of Texas McGovern Medical School at Houston Committee for the Protection of Human Subjects.

## RESULTS

### Demographics and Other Respondent Characteristics

We administered the survey to 928 APSA members, and 353 responded (38% response rate). Most operated primarily within a free-standing children’s hospital (49.7%) or in a children’s hospital within an adult medical center (38.1%). Most respondents were also in an academic (65.3%) or mixed (25.3%) practice, with 9.4% in private practice. The majority of respondents (56.7%) reported holding leadership positions at their institutions, with 43.4% in education and 21.0% in safety positions. Respondents self-reported a median of 13 years in practice since fellowship (interquartile range, 5–17 years).

### Participation in External Safety Programs

**Fig. 1.**
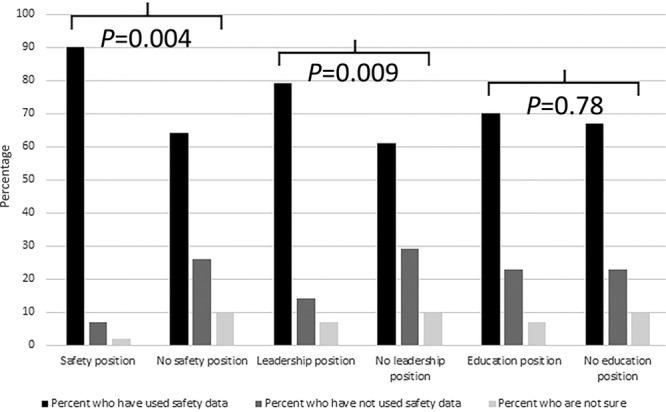
Associations between positions held and use of safety data.

Most respondents (72%) stated that their institution participated in an external patient safety program, such as NSQIP or Solutions for Patient Safety, and 68% reported that they had used information from these programs to improve or monitor patient safety. Those who held leadership or safety positions were more likely to have used data from safety programs (Fig. 1). There was no association between the number of years since graduation from fellowship, type of practice, or type of hospital and the likelihood of using external safety program information. Although there was no quantitative association between private versus academic practice and the likelihood of using safety data, 1 respondent observed “Just changed hospitals recently from academic to private hospital setting. Very different environments.”

The most common reason cited for not using external safety data was “I don’t know much about it but would be interested in learning more” (47.5%). Lack of time (42.9%) and resources (30.4%), as well as difficulty obtaining (21.4%) or interpreting data (19.6%), were also commonly cited barriers. A minority of respondents did not have an interest in the data (3.6%) with a larger proportion thinking the data were not useful (28.6%). One respondent posed the question: “How do I effectively use generic institutional data to help make individual patient care decisions, or to apply that data to monitor my patients’ outcomes?” Another described cost as a barrier to effective use of data: “Doing this right involves hiring/assigning a full-time position to track the data, and I think that cost is one of the larger barriers.”

For those whose institutions do not participate in external safety programs, the main barriers cited were that it was too expensive (40%) or not valued by leadership (15%). As 1 respondent explained, “We were a participant, but our funding was cut by leadership who did not know enough about the program to understand its value.” Another respondent described the challenge of advocating for resources for children’s surgery when working in an adult-focused system: “Participation in national children’s programs has been primarily for ‘Pediatrics’ which has budget capabilities for this, w/o such opportunity for Children’s surgery which is not appropriately organized or valued in our tertiary/quaternary facility that focuses at present more on adults.”

## DISCUSSION

We found that approximately three-quarters of pediatric surgeons practice in institutions that participate in external patient safety programs. Only two-thirds of those surgeons have used these data to drive quality improvement. Despite the ubiquitous promotion of external safety programs in surgical care and demonstrated benefits, the optimal participation and utilization of validated, risk-adjusted benchmark data appear to be suboptimal in pediatric surgery.

National databases and clinical registries are used to compare similar institutions after appropriate risk adjustment. When leveraged properly, this information can improve patient safety, quality of care, and clinical outcomes. Data from external safety programs may be used in combination with individual adverse event review typically performed during morbidity and mortality (M&M) conference to understand trends and inform quality improvement efforts. There is a growing body of literature, including in pediatric surgery, which compares the value of M&M conferences to standardized outcomes databases, such as NSQIP, as tools to measure M&M to drive quality improvement.^6–8^ Surgical M&M relies on voluntary reporting and as such are subject to inadequate and biased sampling.^9^ Surgeons might be unwilling to report all of their complications for fear of ridicule by their peers, combined with fear of litigation and possible institutional constraints, namely a “shame and blame” ethos.^10^ In a single institution retrospective comparison of NSQIP with traditional M&M conference over a 1-year period, NSQIP identified 143 patients with complications as opposed to 58 in M&M. The NSQIP also identified more postoperative deaths and a higher proportion of surgical-site infections and readmissions. Another study that compared NSQIP-Pediatric (NSQIP-P) with surgeon-reported complications at M&M found that M&M captured only 33% of the complications and 83% of the deaths reported in NSQIP-P.^11^ Surgical resident reporting also appears to capture pediatric perioperative complications inadequately when compared with the NSQIP-P database. In a 1-year study in a single institution, residents reported 27 complications, as compared to 68 by NSQIP-P. In particular, resident reporting missed more common sources of postoperative morbidity such as surgical-site infection and transfusion.^12^

Databases such as NSQIP are more reflective of the entire institutional experience. NSQIP relies on a trained third party who systematically reviews a sample of charts according to standard definitions that eliminates the subjectivity inherent in voluntary reporting. The system samples cases and excludes other cases seen by surgical services based on current procedural terminology codes (such as trauma) and nonoperatively managed patients and, therefore, is not reflective of the entire surgical population.^1^ Participation in NSQIP is not, in and of itself, sufficient to decrease adverse events; rather, each hospital must develop in-house programs to act upon NSQIP.^2^ NSQIP is meant to serve as a catalyst for quality improvement, and because the NSQIP program is system-based, the data it provides are conducive to the formation of multidisciplinary groups to study problems and implement change. Action plans must be created and implemented to produce sustainable changes.^13^

Both M&M conference and NSQIP (as well as other external safety programs) are limited in their ability to track outcomes because M&M generally does not capture all complications,^9^ and NSQIP samples only a portion of cases that are performed at a participating institution. An approach to adverse event review that blends M&M and NSQIP can harness and integrate the benefits of both systems.^6^

Surgical M&M is an ideal forum in which to report NSQIP outcomes data.^8^ Real-time event review through traditional M&M can be supplemented by NSQIP event auditing, which is often available at the patient level between 45 and 60 days following surgery and can provide longer term context for individual complications. Furthermore, the M&M process should be closely aligned with other sources of internal event data that have relevance for the care of the pediatric surgical patient. These include Solutions for Patient Safety auditing of hospital-acquired infections, complementary registry data involving perioperative care (eg, Wake Up Safe), and each hospital’s own internal serious adverse event reporting system. Combining M&M, external program data review, and other sources of internal event data can facilitate the development of strategies to identify and reduce the likelihood of preventable adverse events and enable institutions to monitor progress longitudinally against defined benchmarks. Furthermore, such a comprehensive strategy would be better able to facilitate interdepartmental event review and focus on multidisciplinary solutions. The relative contribution of these resources to facilitate comprehensive event review are likely to be different across institutions and may require a coordinated effort between surgeons and other stakeholders (eg, quality administration and representatives from other disciplines) to optimize the process.

**Fig. 2.**
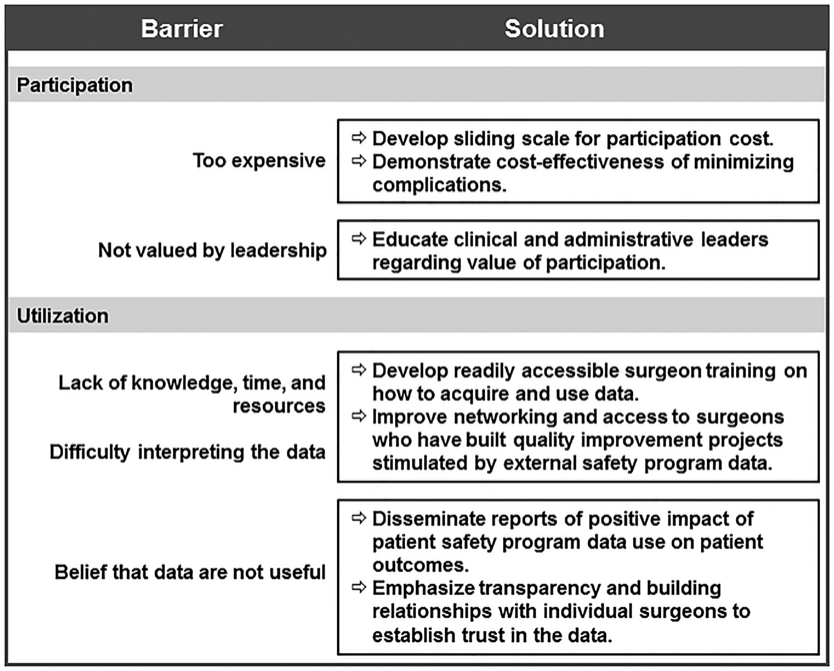
Barriers to use of external safety data and proposed solutions.

It is well known that there are limitations to participation and utilization of data from external safety programs. This study is the first to collect data from surgeons to identify barriers to optimal implementation. We surveyed only 1 group of stakeholders but identified broad themes that may translate to other groups. We summarize these barriers and propose solutions in Figure 2.

## CONCLUSIONS

In 2015, the National Patient Safety Foundation made a compelling argument toward substantial advances in patient safety. They stated that there needs to be greater emphasis on implementation science.^14^ Improving patient safety requires accurate and reliable outcome measurement, understanding of the environment that leads to errors, and rigorously planned implementation of evidence-based interventions.^15^ For pediatric surgeons to effectively utilize data from external patient safety programs, barriers to participation must be addressed. These survey results also demonstrate a clear need for education on how to use safety program data to develop quality improvement projects, and how to effectively engage stakeholders in creating sustainable change.

## DISCLOSURE

The authors have no financial interest to declare in relation to the content of this article.
